# Awareness and Perception of Plastic Surgery among Healthcare Professionals in Pune, India: Do They Really Know What We Do?

**DOI:** 10.1155/2012/962169

**Published:** 2012-05-17

**Authors:** Nikhil Panse, Smita Panse, Priya Kulkarni, Rajendra Dhongde, Parag Sahasrabudhe

**Affiliations:** ^1^Department of Plastic Surgery, B.J Medical College and Sassoon Hospital, Pune 411001, India; ^2^Department of Psychiatry, Maharashtra Institute of Mental Health, Pune 411001, India; ^3^Department of Preventive and Social Medicine, B.J Medical College and Sassoon Hospital, Pune 411001, India; ^4^Department of General Surgery, B.J Medical College and Sassoon Hospital, Pune 411001, India

## Abstract

*Purpose*. The aim of this study is to understand the level of awareness and knowledge of plastic surgery in healthcare professionals in a tertiary health care facility in Pune, India. This study also aims to highlight the perception of the medical professionals about plastic surgery and what they think a plastic surgeon does. *Materials and Methods*. A questionnaire-based survey was done at B.J Medical College and Sassoon Hospital, Pune in 2011. Feedback evaluation forms from hundred resident doctors and faculty were evaluated and analyzed. *Results*. There is not much awareness about plastic surgery as a specialty amongst health care providers. Plastic surgery is mostly perceived as cosmetic surgery, and the other spectrum of the patients we cater to goes largely unnoticed. Of all the clinical conditions given to the participants, there was not a single clinical condition where the respondents favored unanimously for plastic surgeons. *Conclusion*. Plastic surgery as a specialty is poorly understood by our medical colleagues, and the onus of creating and improving the awareness and perception of our specialty lies on us. Herculean unified efforts at individual as well as global level will help us achieve this goal.

## 1. Introduction

 People in today's world are more health conscious and are aware of the different medical specialties. Despite the tremendous advancements in the field of plastic surgery, there seems to be a limited knowledge among the general public and also among medical professionals regarding the spectrum of plastic surgery. As a medical specialty, plastic surgery is poorly understood by both the general public and some medical professionals as well.

This study is an attempt to establish the knowledge of the medical community on the specialty of plastic surgery and the spectrum of patients they cater to.

Unlike in western countries, the public literacy level and awareness in India are very disproportionate. Therefore we did not include the general public in the study. Instead, we included the medical professionals in our study. These people contribute considerably to the health care of the public. We felt that understanding the attitude and perception of our medical colleagues will be more vital than assessing the knowledge of the public. We wanted to know whom should we educate first—the doctors or the public. 

## 2. Materials and Methods

We conducted a questionnaire-based study among a selected group of healthcare professionals to assess their attitude, knowledge, and perception of plastic surgery. A well-structured questionnaire was administered to this group of select individuals, and responses were sought and analysed. The questionnaire was handed over to a total of 100 residents and faculty of nonsurgical specialties. Doctors from general surgery and surgical super specialties, orthopedics, ENT, and ophthalmology were excluded from the study. Dermatology department was also excluded from the study because of the overlapping spectrum of plastic surgery and dermatology. Only M.B.B.S and higher qualification doctors were included in the study.

This survey was conducted at B.J Medical College and Sassoon Hospital, Pune. B.J Medical College and Sassoon Hospital is the largest government tertiary care teaching hospital in Pune. Sassoon General Hospital is 1200 plus bedded with almost 100% bed occupancy round the year. There is a functioning plastic surgery unit in the college.

A questionnaire was designed in 2 parts. In the first part, the participants were asked specific questions pertaining to plastic surgery with a multiple choice option for marking the answers. see Table 1. 

 In the second part, the participants were given a list of clinical situations including trauma, pathology, reconstructive, and cosmetic surgery and were asked to indicate which specialty they think would be the best to treat the clinical situation mentioned. The options of treating surgeons were ENT surgeon, plastic surgeon, ophthalmic surgeon, neurosurgeon, general surgeon, orthopedic surgeon, pediatric surgeon, uro surgeon, oral and maxillofacial surgeon, dermatologist and others (Table 2). 

## 3. Results

The responses of the participants are shown in Tables 3 and 4. Analysis of part 1 of questionnaire showed that 96% of the participants were aware of what training is required to be a plastic surgeon. 12% of participants felt that plastic surgery and cosmetic surgery are the same, and 80% felt that cosmetic surgery is a part of plastic surgery. Of the 100 participants, 83% did not know why plastic surgery is called plastic surgery, 5% felt that it is called plastic surgery because it involves use of plastic, and 4% felt that it is called plastic surgery because face looks shiny like plastic after the surgery. 8% of participants gave various answers of which the nearest relevant one mentioned that plastic was moldable, and in plastic surgery face is moldable, so it is called plastic surgery. A whopping 74% of the participants felt that there are no scar marks left after plastic surgical procedure. 37% of the participants felt that plastic surgery is a very expensive affair and meant for the rich and famous. 87% of participants felt that the risk involved with plastic and cosmetic surgery is similar to other surgeries.

**Figure 1:**
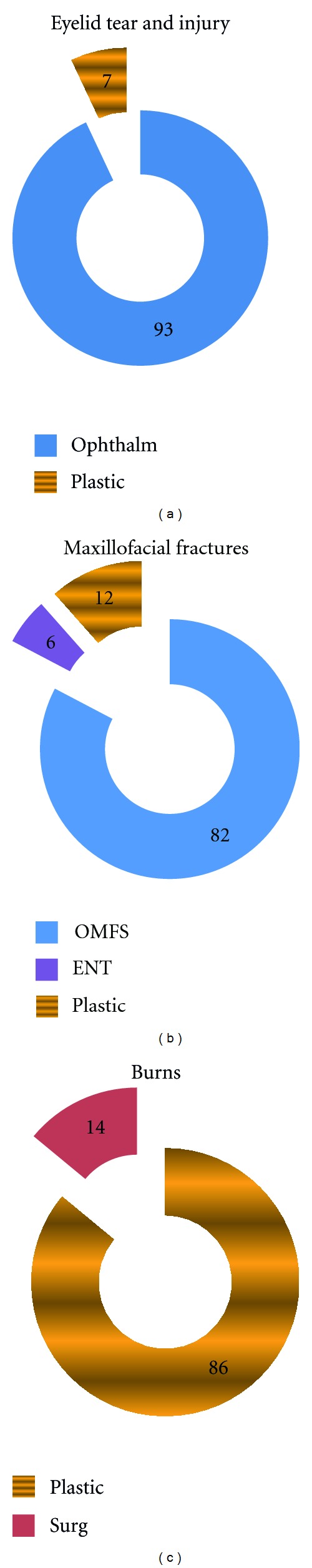


Analysis of part 2 of the questionnaire showed that 93% of respondents preferred an ophthalmic surgeon to suture an eyelid injury, and 92% of respondents preferred a plastic surgeon to suture a cut over the face. When treating a maxillofacial fracture, 82% preferred an oral and maxillofacial surgeon, and 12% preferred a plastic surgeon (Figure 1).

**Figure 2:**
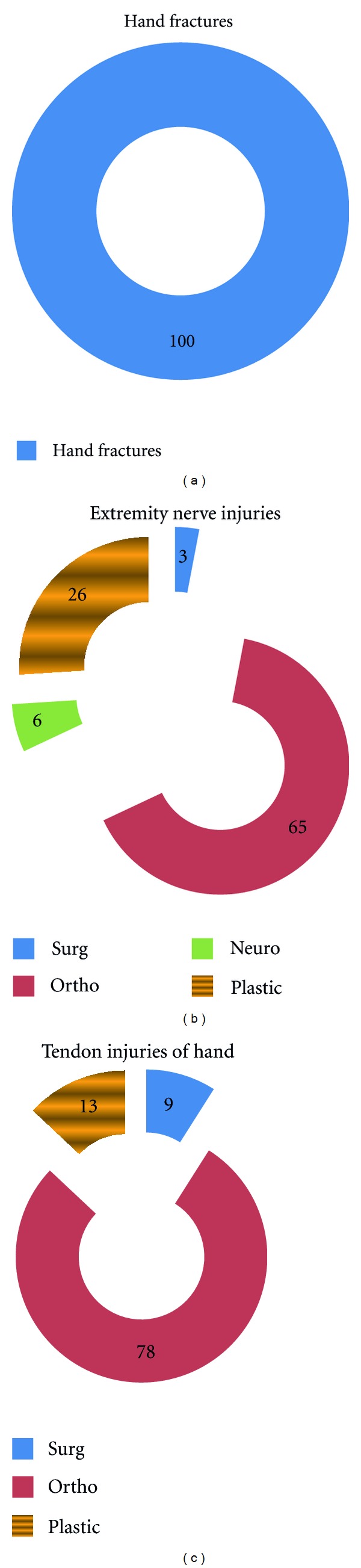


 Most of the respondents (87%) preferred general surgeons over plastic surgeons in management of bed sores. 100% of the participants preferred an orthopedic surgeon to manage a hand fracture. 65% of the participants felt that injuries to nerves of hand and legs must be managed by orthopedic surgeon, 26% felt that they must be managed by a plastic surgeon, and a surprising 6% felt that nerves are best dealt by a neurosurgeon (Figure 2).

 Hypospadias was divided into general surgeons (10%), pediatric surgeon (38%), urosurgeon (26%), and plastic surgeon (26%). Of the other congenital anomalies, 55% felt that plastic surgeons were best to manage a cleft, and 58% felt plastic surgeons were the best to manage congenital ear anomalies. 86% respondents preferred a plastic surgeon for managing burns, and 97% preferred a plastic surgeon for management of postburn deformities. Only 13% of participants preferred plastic surgeon as compared to 78% (orthopedic surgeon) in management of tendon injuries of the hand. The aesthetic surgery procedures have a favorable response towards plastic surgery. 99% participants favored a plastic surgeon doing a liposuction and breast reduction or augmentation procedure. 7% favored a general surgeon doing an abdominoplasty as compared to 93% favoring a plastic surgeon.

**Figure 3:**
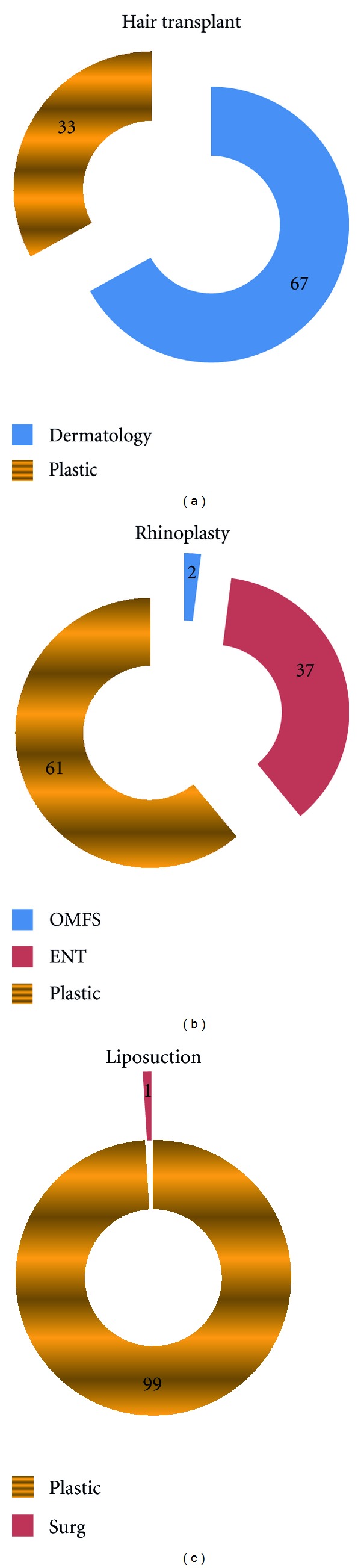


 Majority of participants responded for plastic surgeon (61%) as compared to E.N.T surgeon (37%) for doing a rhinoplasty. Hair transplantation procedure had 67% participants responding for dermatologist as compared to 33% for plastic surgeons (Figure 3). Nonsurgical aesthetic procedures like Botox had 52% respondents favoring dermatologist as compared to 41% for plastic surgeons

The detailed analysis of the responses is shown in Table 4.

Although this survey was done in Pune city in Western India, we believe that the scenario in any part of India and most of the developing world would not be different. Most of our colleagues of other specialties are not really aware of what we are doing. Apart from a few aesthetic surgery procedures, where majority of the respondents favored plastic surgeons, the results for other procedures are disappointing. It was even sad to note that there was not a single clinical condition which participants thought is the exclusive domain of the plastic surgeon. Our specialty is still searching for its identity rather among the healthcare providers than among the general public.

## 4. Discussion

Plastic surgery is a unique specialty that defies definition, has no organ system of its own, and is based on principles rather than specific procedures.

Unlike other medical disciplines, plastic surgery is not defined by an anatomic area (OB/Gyn, ENT, thoracic surgery), organ system (gastroenterology, urology), or patient age group (pediatrics, adolescent medicine, geriatrics). It deals with everything from head to toe but is associated with the prefix plastic which symbolizes nothing to the common man and medical community at large.

There have been surveys on awareness of facial plastic surgery in general population [1], but to our knowledge survey of knowledge and perception of plastic surgery in healthcare professionals is unheard of in our part of the globe.

After analyzing the results of the survey conducted by us, we found that the findings were alarming. It was amply clear that there is not much understanding of our specialty in members of the medical community, and plastic surgery is poorly understood. We feel that if our colleagues from other specialties are not aware of the spectrum of work we do, we ourselves are to be blamed for it. We lag behind in the field of advertisement and awareness creation of our specialty.

At our unit, we have been regularly presenting our work at college-level conferences. We have also been organizing multidisciplinary symposiums of topics like wounds and burns at the local level. In spite of that, the awareness levels of our specialty were very low. To address this problem at our level, we made an activity report comprising the clinical and academic work we have done in the past two years with colour clinical photographs and distributed it amongst all the departments of our college. E-mail addresses were collected of the entire staff and as many residents and students as possible, and soft copies of the activity report were mailed to them all for wider coverage. Copies were also mailed to staff of other medical colleges in Pune.

People have a short memory, and we need to keep on reminding them of our existence. Keeping this in mind, we plan to make brochures of various subspecialties of plastic surgery like pediatric plastic surgery, hand and microsurgery, maxillofacial surgery, burns, and so forth and distribute it for awareness purpose.

We all understand the role the media plays in informing and educating the public on issues of the day, but it seems that when it comes to plastic surgery, the press has a one-track mind that leads them to cosmetic surgery. Plastic surgeons are often portrayed in the media as glamorous beings who give people a new lease of life through a “nip and a tuck”. However, the reconstructive side of our work goes largely unreported. As Reid and Malone [2] highlight, of 1191 articles published in British newspapers in 2006, 89% used the term “plastic surgery” in the context of cosmetic surgery, with only 10% referring to reconstructive work. There has been a dramatic increase in the number of people having cosmetic surgery in India over the last few years. The International Society of Aesthetic Plastic Surgery (ISAPS) has produced the ISAPS Biennial Global Survey (TM) of plastic surgeons and procedures in the top 25 countries and regions [3]. They say this ISAPS survey marks the first time reliable international plastic surgery data which has been obtained and analyzed by independent statistical specialists. India ranks fourth in the list and is emerging as a major centre of cosmetic surgery [3].

This in itself means that cosmetic surgery is a popular topic of discussion, so it is little wonder that the media want to cover it. However, no part of reconstructive aspect of plastic surgery is covered by the media. For all of us as plastic surgeons, plastic surgery is not just cosmetic and we need to educate and inform the public and nonplastic surgical specialties as to what it is that plastic surgeons actually do. Park et al. [4] looked at perception and knowledge around the work of plastic surgeons. They found that 23.7% of the local lay population could not think of five conditions treated by plastic surgeons, while 27% felt that the majority of the work plastic surgeons did was cosmetic in nature. It is not just the general public who has this perception. Journalists were interviewed across the national press, from The Times, The Daily Mail, and BBC News Online and it was found that journalists catering for a consumer audience feel that plastic surgery is synonymous with cosmetic surgery [5].

There are numerous facets to it and lot of attempts need to be made at an individual, local, regional, national, and global level to bring about this change effectively.

After analysis of the results, we realized that the name of our specialty itself is not understood by many. Unlike other specialties, where the name itself indicates the work being done by that particular specialty, plastic surgery lags behind on this front. In India, it is not uncommon for us to encounter patients who after completion of all the procedures will ask when plastic surgery will be done and plastic will be used ! We need to give serious thoughts about changing the name of our specialty to plastic, reconstructive and aesthetic surgery and name of our respective associations from Association of Plastic Surgeons to Association of Plastic, Reconstructive, and Aesthetic Surgeons. Similarly, journal names of respective bodies can be changed to journal of plastic, reconstructive, and aesthetic surgery of the respective associations. It would be an important first step in trying to improve understanding of what plastic surgeons do. It would help to change the mindset of peers and members of the public.

A similar effort was done by the British Association of Plastic Surgeons (BAPS), who changed the name of the organization to British Association of Plastic, Reconstructive and Aesthetic Surgeons (BAPRAS) in July 2006 [5].

We feel that one of the important reasons for poor knowledge and perception of plastic surgery in our survey is inadequate training and exposure of plastic surgery at undergraduate level. There has been much discussion in the western literature regarding if there is a place for plastic surgery in the undergraduate curriculum. As outlined by Wade et al. [6] many undergraduates are in favor of having plastic surgery teaching even though many may not necessarily want to pursue a career in the specialty. The portrayal of plastic surgery in the media is frequently that of purely elective cosmetic operations and this is often the understanding that undergraduates have of the Specialty until they do further research and discover the main workload carried out by a plastic surgeon. Undergraduate exposure is the single most influential factor for subsequent career interest in plastic surgery [7] with the duration of specialty exposure directly proportional to subsequent career intentions [8]. Given the inherent difficulties of changing our university curriculum, a simple but effective solution to increase plastic surgery exposure to all our medical students is required. A career in plastic surgery day has been held at the Royal College of Surgeons in London with talks from surgeons in different subspecialties and on careers in the profession, in addition to surgical skills practical sessions under the supervision of trainees in the profession [9]. An attempt to introduce a similar module can be made at other places as well. These conferences can give students a good insight into the specialty. Teaching undergraduate plastic surgery has potential benefits to all future doctors and ultimately patients, irrespective of career intentions. Exposure to plastic surgery may not lead to the development of a career interest in plastic surgery [9]. However, this is still an important reason to encourage undergraduate teaching, as a negative decision allows students to choose more suitable career paths. Given the multidisciplinary nature of plastic surgery and the abundant team working with other medical and surgical specialties, it is important that nonplastic surgery colleagues are aware of the work carried out by plastic surgeons. Referrals will therefore be more accurate, resulting in a more efficient and higher quality of service delivered to patients [9].

 An attempt should be made by all of us to include in the undergraduate teaching, a topic on the spectrum of plastic surgery with clinical photographs to impress upon the young minds, as to what plastic surgery is all about. We want to attract the best and brightest of medical students into our specialty, so as a group, plastic surgeons can continue to be at the forefront in medical advances. This can be done only by creating awareness of the breadth of the plastic surgeons role and the contribution of our specialty to patient care.

Telemedicine can be an important tool for educating the medical practitioners at remote areas. Teleeducation of the doctors at remote areas about the spectrum of plastic surgery will also result in more accurate and direct referrals for our specialty.

For wider coverage and public awareness, help of media must be sought. It is necessary that we as plastic surgeons give ample information to the media regarding the reconstructive aspects of plastic surgery. Professional communication services can be hired if needed to improvise on communication and awareness. Celebrities can be requested or hired to focus on the reconstructive part of plastic surgery. Innovative ways of reaching the public must be sought and implemented.

Individuals and national-level organizations should make effective efforts for campaigns to educate the health care consumers and providers and for projects to publicize the specialty of plastic surgery. Awareness of our medical colleagues could be improved by arranging more interdisciplinary sessions and interdepartmental discussions. We feel that national-level organizations are after all a conglomeration of individuals of the specialty. Efforts from each and every individual will make a difference.

To conclude, plastic surgery as a specialty is poorly understood by our medical colleagues, and the onus of creating and improving the awareness and perception of our specialty lies on us. Herculean unified efforts at individual as well as global level will help us achieve this goal. The future of plastic, reconstructive, and aesthetic surgery is bright, and we can make it even brighter by our collective efforts in the years to follow.

## Figures and Tables

**Table 1:** 

Request you to please spare five minutes of your time to fill up this form. Please answer all the questions.	
Name (optional):	
Educational qualification: Department:	
Email (optional-preferable): Mobile (optional):	
Questionaire:	
(1) What is the training required to be a Plastic surgeon?	
(a) Three years training in General surgery after M.B.B.S followed by three years training in Plastic surgery.	
(b) Six years training after B.A.M.S or B.H.M.S.	
(c) Both of the above.	
(d) Don't know.	
(2) Do you feel Plastic surgery and Cosmetic surgery are the same	
(a) Yes.	
(b) No.	
(c) Cosmetic surgery is a part of plastic surgery.	
(d) Don't know.	
(3) Why do you think Plastic surgery is called “Plastic” surgery?	
(a) It involves use of Plastic in surgery.	
(b) After surgery the face looks shiny like plastic..	
(c) Don't know.	
(d) Any other reason. Please specify…………………………	
(4) Do you think that after a plastic surgery operation, there are scar marks left over face?	
(a) Yes, there are scar marks.	
(b) No, there are no Scar marks.	
(c) Don't know.	
(5) Do you think that plastic surgery is a very expensive affair and meant for rich and famous?	
(a) Yes.	
(b) No.	
(c) Don't know.	
(6) Do you think plastic and cosmetic surgeries are very risky?	
(a) They are very risky.	
(b) The risk is similar to risk involved in other surgeries.	
(c) Does not involve any risk.	
(d) Dont know.	

**Table 2:** Which surgeon would you expect to treat the following conditions?

Sr. no.	Ailment	General surgeon	Orthopaedic surgeon	Neuro- surgeon	Oral and maxillofacial surgeon	ENT surgeon	Skin specialist	Pediatric surgeon	Uro- surgeon	Ophthalmic surgeon	Plastic surgeon	Others
(1)	Eyelid tear and injury											
(2)	Fracture of the jaw and face											
(3)	Bed sore											
(4)	Diabetic foot wounds											
(5)	Hair transplantation											
(6)	Cleft lip and palate (congenital)											
(7)	Cuts over the face											
(8)	Fractures of Hand											
(9)	Difficulty in opening mouth											
(10)	Injury to nerves of hand and legs											
(11)	Deformities of leprosy											
(12)	Rhinoplasty (nose Job)											
(13)	Hypospadias (congenital deformity of penis)											
(14)	Totally amputed thumb, finger, or hand											
(15)	Burns											

(16)	Congenital anomalies of ear and nose											
(17)	Abdominoplasty (tummy tuck)											
(18)	Botox											
(19)	Burn deformities like crooked hands, and so forth											
(20)	Liposuction (fat aspiration)											
(21)	Sex change surgery.											
(22)	Vitiligo (white patches)											
(23)	Surgery for facial wrinkles											
(24)	Non healing wounds over legs											
(25)	Tendon injuries of hand											
(26)	Breast reduction or enhancement surgery											
(27)	Skin grafting											
(28)	Electrical burns											

**Table 3:** 

Request you to please spare five minutes of your time to fill up this form. Please answer all the questions.		
Name (optional):		
Educational qualification:	Department:	
Email (optional-preferable):	Mobile (optional):	
Questionaire:		

(1) What is the training required to be a plastic surgeon?		

Sr. no.	Question	Reply-Total 100

(a)	Three years training in general surgery after M.B.B.S followed by three years training in plastic surgery.	96
(b)	Six years training after B.A.M.S or B.H.M.S.	0
(c)	Both of the above.	2
(d)	Do not know	2

(2) Do you feel plastic surgery and cosmetic surgery are the same		

Sr. no.	Question	Reply-Total 100

(a)	Yes.	12
(b)	No.	4
(c)	Cosmetic surgery is a part of plastic surgery	80
(d)	Do not know	4

(3) Why do you think Plastic surgery is called “plastic” surgery?		

Sr. no.	Question	Reply-Total 100

(a)	It involves use of Plastic in surgery	5
(b)	After surgery the face looks shiny like plastic	4
(c)	Do not know	83
(d)	Any other reason. Please specify…………	8

(4) Do you think that after a plastic surgery operation, there are scar marks left over face?		

Sr. no.	Question	Reply-Total 100

(a)	Yes, there are scar marks.	19
(b)	No, there are no scar marks.	74
(c)	Do not know	7

(5) Do you think that plastic surgery is a very expensive affair and meant for rich and famous?		

Sr. no.	Question	Reply-Total 100

(a)	Yes	37
(b)	No.	55
(c)	Do not know	8

(6) Do you think plastic and cosmetic surgeries are very risky?		

Sr. no.	Question	Reply-Total 100

(a)	They are very risky.	7
(b)	The risk is similar to risk involved in other surgeries	87
(c)	Does not involve any risk	5
(d)	Do not know.	1

**Table 4:** Which surgeon would you expect to treat the following conditions?

Sr. no.	Ailment	General surgeon	Orthopaedic surgeon	Neuro surgeon	Oral & Maxillofacial surgeon	ENT surgeon	Skin specialist	Pediatric surgeon	Uro surgeon	Ophthalmic surgeon	Plastic surgeon	Others
(1)	Eyelid tear and injury									93	**7**	
(2)	Fracture of the jaw and face				82	6					**12**	
(3)	Bed sore	87									**13**	
(4)	Diabetic foot wounds	84	8								**8**	
(5)	Hair transplantation						67				**33**	
(6)	Cleft lip and palate (congenital)				16	3		26			**55**	
(7)	Cuts over the face.	8									**92**	
(8)	Fractures of Hand.		100									
(9)	Difficulty in opening mouth.				41	54					**5**	
(10)	Injury to nerves of hand and legs.	3	65	6							**26**	
(11)	Deformities of leprosy.		44				4				**52**	
(12)	Rhinoplasty (nose job)				2	37					**61**	
(13)	Hypospadias (congenital deformity of penis)	10						38	26		**26**	
(14)	Totally amputed thumb, finger, or hand.	3	43								**54**	
(15)	Burns	13					1				**86**	

(16)	Congenital anomalies of ear and nose					36		6			**58**	
(17)	Abdominoplasty (tummy tuck)	7									**93**	
(18)	Botox		7				52				**41**	
(19)	Burn deformities like crooked hands, and so forth		3								**97**	
(20)	Liposuction (fat aspiration)	1									**99**	
(21)	Sex change surgery.	2							50		**48**	
(22)	Vitiligo (white patches)	3					71				**26**	
(23)	Surgery for facial wrinkles						56				**44**	
(24)	Non healing wounds over legs	69	15								**16**	
(25)	Tendon injuries of hand	9	78								**13**	
(26)	Breast reduction or enhancement surgery.	1									**99**	
(27)	Skin grafting	24									**76**	
(28)	Electrical burns	17									**83**	
